# Structural correlates of attention dysfunction in Lewy body dementia and Alzheimer’s disease: an ex-Gaussian analysis

**DOI:** 10.1007/s00415-019-09323-y

**Published:** 2019-04-21

**Authors:** Julia Schumacher, Ruth Cromarty, Peter Gallagher, Michael J. Firbank, Alan J. Thomas, Marcus Kaiser, Andrew M. Blamire, John T. O’Brien, Luis R. Peraza, John-Paul Taylor

**Affiliations:** 1 Institute of Neuroscience, Newcastle University, Biomedical Research Building 3rd Floor, Campus for Ageing and Vitality, Newcastle upon Tyne, NE4 5PL UK; 2 Institute of Neuroscience, Newcastle University, The Henry Wellcome Building, Newcastle upon Tyne, NE2 4HH UK; 3 Interdisciplinary Computing and Complex BioSystems (ICOS) Research Group, School of Computing, Newcastle University, Newcastle upon Tyne, NE4 5TG UK; 4 Institute of Cellular Medicine and Newcastle Magnetic Resonance Centre, Campus for Ageing and Vitality, Newcastle upon Tyne, NE4 5PL UK; 5 Department of Psychiatry, University of Cambridge School of Medicine, Cambridge, CB2 0SP UK

**Keywords:** Attention network test, Cognitive fluctuations, Voxel-based morphometry, Parkinsonism, Neurodegeneration

## Abstract

**Background:**

Lewy body dementia (LBD) and Alzheimer’s disease (AD) are common forms of degenerative dementia. While they are characterized by different clinical profiles, attentional deficits are a common feature. The objective of this study was to investigate how attentional problems in LBD and AD differentially affect different aspects of reaction time performance and to identify possible structural neural correlates.

**Methods:**

We studied reaction time data from an attention task comparing 39 LBD patients, 28 AD patients, and 22 age-matched healthy controls. Data were fitted to an ex-Gaussian model to characterize different facets of the reaction time distribution (mean reaction time, reaction time variability, and the subset of extremely slow responses). Correlations between ex-Gaussian parameters and grey and white matter volume were assessed by voxel-based morphometry.

**Results:**

Both dementia groups showed an increase in extremely slow responses. While there was no difference between AD and controls with respect to mean reaction time and variability, both were significantly increased in LBD patients compared to controls and AD. There were widespread correlations between mean reaction time and variability and grey matter loss in AD, but not in LBD.

**Conclusions:**

This study shows that different aspects of reaction time performance are differentially affected by AD and LBD, with a difference in structural neural correlates underlying the observed behavioural deficits. While impaired attentional performance is linked to brain atrophy in AD, in LBD it might be related to functional or microstructural rather than macrostructural changes.

**Electronic supplementary material:**

The online version of this article (10.1007/s00415-019-09323-y) contains supplementary material, which is available to authorized users.

## Introduction

Lewy body dementia (LBD), which includes both dementia with Lewy bodies (DLB) and Parkinson’s disease dementia (PDD), represents the second to third most common form of neurodegenerative dementia in older age after Alzheimer’s disease (AD) [1]. DLB and PDD show a similar clinical profile characterized by complex visual hallucinations, cognitive fluctuations, and Parkinsonian motor symptoms [2, 3] and are only differentiated by whether the cognitive or the motor symptoms occur first [4].

Attentional and executive deficits occur frequently in both AD and LBD [5]. Reaction times have been shown to be increased and more variable in patients with dementia in general, and LBD patients show slower reaction times and higher intraindividual variability than AD patients [6, 7]. This difference increases with increasing task demand, especially when the task involves an executive or spatial aspect [7]. Additionally, LBD patients with more marked cognitive fluctuations have slower reaction times, impaired vigilance and higher fluctuations in reaction time performance compared to those patients with less severe fluctuations [8–10].

A large majority of reaction time studies focus on analysing mean reaction times. However, reaction times are usually not normally distributed, but positively skewed. Describing this distribution by more than central tendency measures, therefore, provides a more detailed and accurate analysis method [11]. One distribution that has been used successfully to model empirical reaction times is the exponentially modified Gaussian (ex-Gaussian) distribution. This is a convolution of a Gaussian and an exponential distribution characterized by three parameters—mu, sigma, and tau—that describe different aspects of the ex-Gaussian distribution. Mu and sigma describe the mean and standard deviation of the Gaussian part, respectively, while tau quantifies the right tail of the distribution which describes the subset of extremely slow responses [12]. The ex-Gaussian analysis, therefore, allows investigation of the effect of ageing and dementia on different aspects of reaction time distributions. An overall shift of the distribution to higher or lower values will be primarily reflected by a change in mu, whereas changes in skewness will be indicated by a change in tau. While ageing has been shown to affect all three parameters, AD only affects the tau component compared to age-matched controls without dementia [13, 14].

Even though attentional dysfunction is a core feature of LBD, no previous study has analysed reaction times in LBD with an ex-Gaussian analysis. The first aim of the present study is, therefore, to investigate how different aspects of reaction times as modelled by the ex-Gaussian distribution are differentially affected in LBD compared to AD and healthy ageing. Based on previous studies, we hypothesized to see an increase in tau in the AD group compared to healthy controls with little change in mu and sigma [13, 14]. Given that attentional impairment is more pronounced in LBD than in AD, we expected to see an increase in all three ex-Gaussian parameters in LBD compared to controls and AD. Tau represents the subset of extremely slow responses and, therefore, specifically captures lapses in attention or attentional fluctuations [15]. Since fluctuations in attention are a core symptom of LBD and less common in AD [16], we hypothesized to see a specific increase in tau in the LBD group compared to AD.

A second aim of this study is to investigate the relationship between the different ex-Gaussian parameters and clinical scores in the LBD group, especially with respect to cognitive fluctuations. The clinical identification of cognitive fluctuations can often be challenging and different studies have underscored the need for more objective markers of fluctuations in LBD [10, 17, 18]. Given the association between tau and attentional fluctuations [15] and evidence from previous studies for a link between cognitive fluctuations and reaction time variability [8–10], we investigated tau as a potential candidate for such an objective marker of fluctuations in LBD.

Previous studies have suggested an association between reaction time deficits and structural brain abnormalities in AD [13]. The third aim of this study is, therefore, to investigate possible macrostructural neural correlates of reaction time deficits in AD and LBD. This was done by analysing voxelwise relations between the three ex-Gaussian parameters and grey and white matter volume in both dementia groups.

## Methods

### Participants

The study involved 103 participants who were over 60 years of age. Patients were recruited from the local community-dwelling population who had been referred to old age psychiatry and neurology services. The study was approved by the local ethics committee. Forty-eight participants were diagnosed with probable LBD (26 DLB and 22 PDD patients), 33 with probable AD, and 22 were age-matched healthy controls (HC) with no history of psychiatric or neurological illness. Clinical diagnoses were performed independently by two experienced old age psychiatrists in alignment with consensus criteria for probable DLB [2], PDD [19], and AD [20]. To aid clinical diagnosis, DAT-scans were performed in a subset of the DLB patients [21, 22].

### Modified attention network test

We used a modified version of the attention network test (ANT) [23, 24] based on the version described by Fan et al. [25]. The main rationale for adapting the ANT was to make it suitable for older adults and dementia patients. This was achieved by increasing the size of the stimuli to account for participants with poor visual acuity and by adjusting the timings to account for slower cognitive processing speed in older adults [26]. A detailed description of the task can be found in Ref. [24]. Briefly, the ANT combines elements of the Erikson flanker task [27] and the Posner cueing paradigm [28] to form a single visual reaction time task. There are three different cue conditions: no cue, neutral cue, or spatial cue. This is followed by a flanker task during which, in our modified ANT, participants are asked to decide upon the majority direction of four arrows. The arrows either all point to the same direction (congruent target) or one arrow points to the opposite direction (incongruent target). Participants completed between 6 and 10 (median = 8) runs of the task, each run comprising 36 trials. All trials from runs with less than 2/3 correct responses were excluded from the analysis as performance below this was not different from chance [23]. Additionally, participants with less than 70 remaining correct trials were excluded to allow a robust fit of the ex-Gaussian distribution.

### Ex-Gaussian analysis

**Fig. 1:**
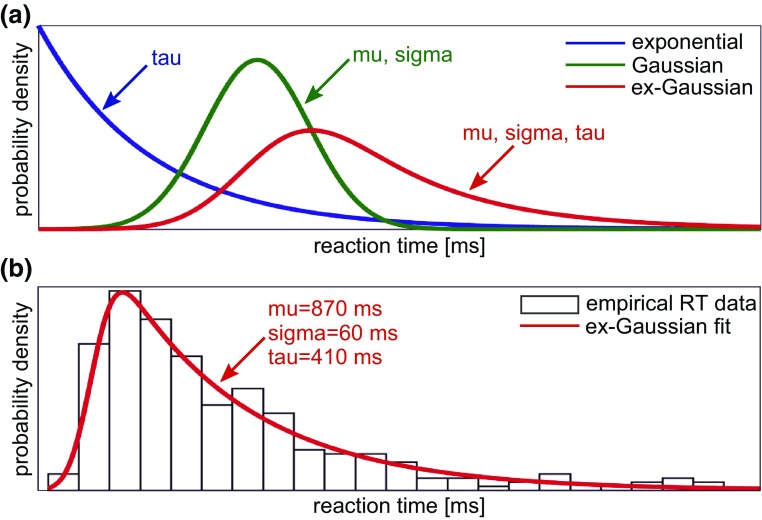
**a** Ex-Gaussian function as a convolution of a Gaussian function with mean mu and standard deviation sigma and an exponential function with decay parameter tau. **b** Example fit of the ex-Gaussian function to an empirical reaction time (RT) distribution

Response times from the ANT were analysed by fitting an ex-Gaussian distribution to the response times from all correct trials for each participant individually (combining all cue and target conditions). The ex-Gaussian distribution is a convolution of a Gaussian and an exponential distribution and can be described by three parameters. Mu and sigma represent the mean and standard deviation of the Gaussian component, respectively. Tau is the decay parameter of the exponential component and characterizes the slow tail of the distribution (see Fig. 1). Ex-Gaussian parameters for each participant were estimated using the DISTRIB toolbox in Matlab (R2015b) which applies a maximum likelihood approach with a bounded search [29].

### VBM analysis

Structural images were acquired on a 3 T Philips Intera Achieva scanner with a magnetization prepared rapid gradient echo (MPRAGE) sequence, sagittal acquisition, echo time 4.6 ms, repetition time 8.3 ms, inversion time 1250 ms, flip angle = 8°, SENSE factor = 2, and in-plane field of view 240 × 240 mm^2^ with slice thickness 1.0 mm, yielding a voxel size of 1.0 × 1.0 × 1.0 mm^3^.

A voxel-based morphometry (VBM) analysis was performed in SPM12 (http://www.fil.ion.ucl.ac.uk/spm/) to assess voxelwise correlations between the ex-Gaussian parameters and cortical volume. This analysis was performed separately in the AD and LBD groups. First, images were segmented into grey matter, white matter, and cerebrospinal fluid. The segmented grey and white matter images were then coregistered and normalized to MNI space using SPM’s DARTEL algorithm [30] and modulated. As a final step, images were smoothed with an 8 mm full width at half maximum Gaussian kernel. Using these images, correlations between grey and white matter volume and the ex-Gaussian parameters were assessed using multiple regression in SPM for each ex-Gaussian parameter separately. Covariates of no interest for age, gender, and total intracranial volume were included in the design matrix. The UPDRS motor score was included as an additional covariate in the LBD group. An explicit mask was estimated using the SPM Masking Toolbox [31] to restrict the statistical analysis to voxels that represent grey and white matter, respectively. An uncorrected voxel-level threshold of *p* < 0.001 was chosen. Subsequently, the minimum cluster size for a corrected threshold of *p* < 0.05 was determined using the 3dClustSim function in AFNI (https://afni.nimh.nih.gov).

### Statistics

Statistical analyses were carried out in IBM SPSS Statistics version 22. Kolmogorov–Smirnov normality tests indicated that the ex-Gaussian parameters were not normally distributed in all clinical groups (*p* < 0.05). Therefore, the three ex-Gaussian parameters were compared between the groups by a non-parametric Kruskal–Wallis ANOVA and post hoc Dunn’s tests with Bonferroni correction. Correlations between the ex-Gaussian parameters and different clinical scores were assessed by Spearman’s rank correlations and *p* values were FDR corrected for multiple comparisons.

## Results

### Demographics

Five AD, three DLB, and six PDD patients were excluded because they did not fulfil the reaction time performance criteria (see “Modified attention network test” section). This resulted in 28 AD, 39 LBD (23 DLB and 16 PDD), and 22 HC participants for further analysis.

Demographic and clinical information for all included participants is presented in Table 1. All three groups were matched for age and gender. As expected, the LBD group had more frequent occurrence of the core LBD symptoms (cognitive fluctuations, visual hallucinations, and Parkinsonism) than the AD group. However, they were slightly less impaired in terms of overall cognition (MMSE and CAMCOG) and the time since the onset of cognitive symptoms was shorter in the LBD group compared to the AD group. To ensure that group differences in overall cognition did not influence the results, all analyses were repeated on AD and LBD subgroups that were matched for overall cognition (see “Comparison of demographics and clinical variables for matched AD and LBD subgroups” section of the Supplementary Material). The percentage of patients taking cholinesterase inhibitors did not differ between the two dementia groups whereas significantly more LBD patients were taking dopaminergic medication. Fourteen DLB patients underwent a DAT-scan; all except one of these patients showed an abnormal scan.

**Table 1:** Demographics and clinical information, mean (standard deviation)

	HC (*N* = 22)	AD (*N* = 28)	LBD (*N* = 39)	Between-group differences
Male:female	15:7	22:6	34:5	*χ*^2^ = 3.18, *p* = 0.20^a^
Age	75.9 (5.4)	76.6 (8.1)	75.5 (5.5)	*F*_2,86_ = 0.22, *p* = 0.80^b^
AChEI	na	26	35	*χ*^2^ = 0.19, *p* = 0.66^c^
PD meds	na	0	28	*χ*^2^ = 34.54, *p* < 0.001^c^
Duration	na	3.9 (2.1)	3.0 (1.9)	*U* = 395, *p* = 0.05^d^
MMSE	29.2 (0.9)	21.8 (3.1)	23.5 (3.7)	*t*_65_ = 1.94, *p* = 0.06^e^
CAMCOG	96.7 (3.7)	71.0 (11.5)	76.2 (12.5)	*t*_65_ = 1.73, *p* = 0.09^e^
UPDRS	1.1 (1.4)	2.1 (2.0)	19.2 (8.4)	*t*_65_ = 10.47, *p* < 0.001^e^
CAF total	na	0.7 (1.7)^f^	5.0 (4.7)	*t*_64_ = 4.46, *p* < 0.001^e^
Mayo total	na	8.8 (4.0)^f^	13.2 (5.9)	*t*_64_ = 3.39, *p* = 0.001^e^
Mayo cogn	na	1.8 (1.8)^f^	2.7 (1.9)	*t*_64_ = 1.96, *p* = 0.05^e^
NPI total	na	6.9 (6.4)^f^	13.3 (10.4)	*t*_64_ = 2.89, *p* = 0.005^e^
NPI hall	na	0.04 (0.2)^f^	1.7 (2.1)	*t*_64_ = 4.14, *p* < 0.001^e^

*AChEI* number of patients taking acetylcholinesterase inhibitors, *AD* Alzheimer’s disease, *CAF total* Clinical Assessment of Fluctuations total score, *CAMCOG* Cambridge Cognitive Examination, *Duration* duration of cognitive symptoms in years, *HC* healthy controls, *LBD* Lewy body dementia, *Mayo cogn* Mayo Fluctuations Cognitive Subscale, *Mayo total*, Mayo Fluctuations Scale, *Mayo arousal* Mayo Fluctuations Arousal Subscale, *MMSE* Mini Mental State Examination, *na* not applicable, *PD meds* number of patients taking dopaminergic medication, *UPDRS* Unified Parkinson’s Disease Rating Scale III, *NPI* Neuropsychiatric Inventory, *NPI hall* NPI Hallucination Subscore

^a^Chi-square test HC, AD, DLB

^b^One-way ANOVA HC, AD, DLB

^c^Chi-square test AD, DLB

^d^Mann–Whitney *U* test AD, DLB

^e^Student’s *t* test AD, DLB

^f^*N* = 27

It was decided a priori to combine the DLB and PDD patients into one Lewy body dementia group as previous studies have shown similar attentional and executive impairment in DLB and PDD [9, 23] as well as similar brain structural abnormalities [32]. The DLB and PDD subgroups were matched in terms of age, overall cognition, and dementia duration (see Supplementary Table S1). PDD patients were more impaired in terms of Parkinsonism, psychiatric symptoms, and cognitive fluctuations, and were more often on dopaminergic medication than DLB patients.

The number of recorded trials did not differ between the three groups (mean HC = 301.1, mean AD = 295.7, mean LBD =  302.8; Kruskal–Wallis ANOVA, *F*_2_ = 1.07, *p* = 0.59). The percentage of correct trials was higher in the control group than in the AD and the LBD group, but did not significantly differ between the two dementia groups (mean HC = 98%, mean AD = 90%, mean LBD = 85%; Kruskal–Wallis test, *F*_2_ = 33.04, *p* < 0.001; post hoc Dunn’s test, *p* (HC, AD) < 0.001, *p* (HC, LBD) < 0.001, *p* (AD, LBD) = 0.27).

### Comparison of ex-Gaussian parameters

**Fig. 2:**
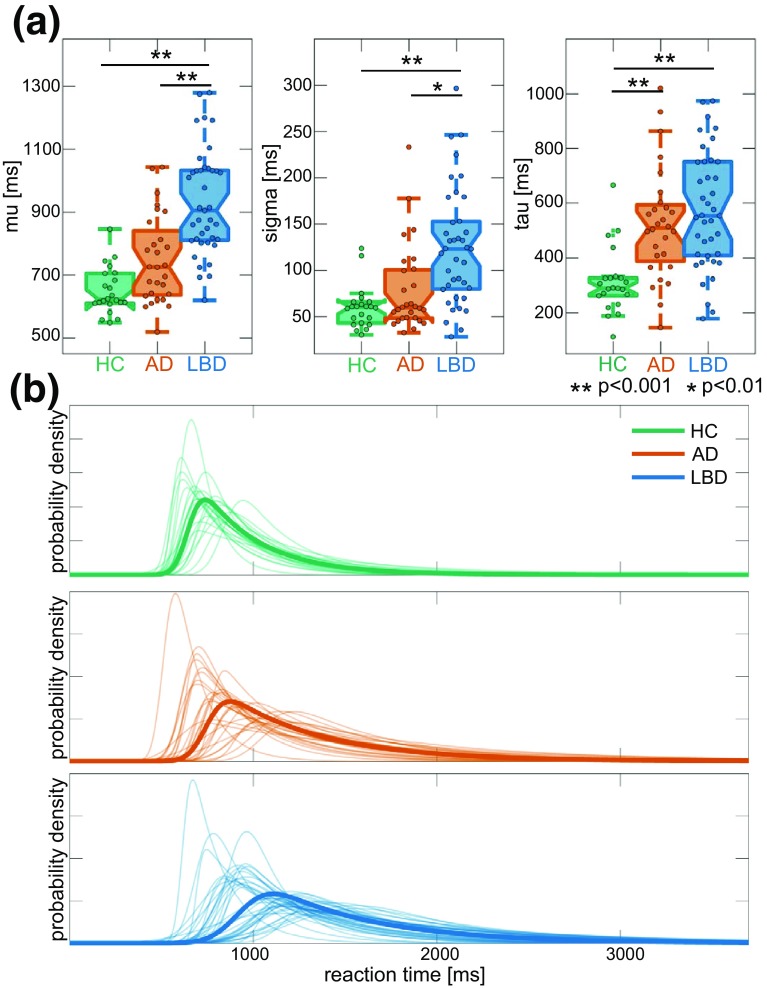
**a** Comparison of ex-Gaussian parameters between HC, AD, and LBD, see Table 2 for more detailed statistics. **b** Fitted ex-Gaussian distributions for mean parameters within each group (thick lines) and for each individual participant (thin lines)

Mu was significantly increased in the LBD group compared to both controls and AD while there was no significant difference in mu between controls and AD. The same effect was observed for sigma. Tau was significantly increased in both dementia groups compared to controls, but there was no significant difference in tau between the two dementia groups (see Fig. 2 and Table 2). These results persisted when analyzing matched dementia subgroups (see Supplementary Table S3).

**Table 2:** Ex-Gaussian parameters, mean (standard deviation)

	HC	AD	LBD	Kruskal–Wallis	Post hoc tests		
					HC vs. AD	HC vs. LBD	AD vs. LBD
Mu	649.43 (73.88)	748.90 (139.38)	930.73 (166.22)	*F*_2_ = 40.49, *p* < 0.001	*p* = 0.06	*p* < 0.001	*p* < 0.001
Sigma	59.85 (22.87)	78.57 (46.98)	125.03 (62.75)	*F*_2_ = 23.25, *p* < 0.001	*p* = 0.88	*p* < 0.001	*p* = 0.001
Tau	313.48 (119.50)	523.28 (202.89)	572.99 (213.21)	*F*_2_ = 24.16, *p* < 0.001	*p* < 0.001	*p* < 0.001	*p* = 1.00

Between-group differences were assessed by Kruskal–Wallis ANOVA with Dunn’s post hoc tests, *p* values are Bonferroni-corrected for multiple comparisons

*AD* Alzheimer’s disease, *HC* healthy controls, *LBD* Lewy body dementia

Supplementary Table S4 shows a comparison of ex-Gaussian parameters when DLB and PDD were treated as separate groups. Overall, the PDD group seemed to be more impaired than the DLB group (higher mu, sigma, and tau); however, none of the differences between DLB and PDD were significant after correcting for multiple comparisons.

### Correlations with clinical scores in the dementia groups

In the LBD group, there was a significant positive correlation between the percentage of correct responses and the MMSE (see Table 3). There was also a trend for a positive correlation between the UPDRS and mu and sigma. However, this correlation did not survive correction for multiple comparisons. There was no significant correlation between any of the ex-Gaussian parameters and any clinical fluctuations score (see Table 3 and Supplementary Table S5).

**Table 3:** Spearman’s rank correlations of Ex-Gaussian parameters and overall performance with clinical scores in the LBD and AD groups separately, correlation coefficient (*p* value, FDR corrected for multiple comparisons)

	LBD			AD
	Mayo cogn	UPDRS	MMSE	MMSE
Mu	0.31 (0.11)	0.34 (0.09)	− 0.10 (0.57)	− 0.59 (0.007)
Sigma	0.29 (0.11)	0.39 (0.06)	0.06 (0.72)	− 0.52 (0.02)
Tau	0.32 (0.10)	0.23 (0.22)	− 0.15 (0.46)	− 0.42 (0.08)
% Correct	− 0.15 (0.46)	− 0.11 (0.57)	0.62 (0.0005)	0.35 (0.11)

*Mayo cogn* Mayo Fluctuations Cognitive Subscale, *MMSE* Mini Mental State Examination, *UPDRS* Unified Parkinson’s Disease Rating Scale III, *% Correct* percentage of correct trials across all included runs

In the AD group, mu and sigma were negatively correlated with the MMSE. Furthermore, there was a trend for a negative correlation between MMSE and tau.

### Correlations with grey and white matter volume in Lewy body dementia

Three DLB and two AD patients did not have structural MRI scans available and were, therefore, excluded from the VBM analysis. When comparing overall grey matter volume between the groups, AD and LBD showed decreases in grey matter volume compared to controls and these deficits were more pronounced in AD than in LBD (see Supplementary Figures S2–S4). There were no differences in grey matter volume between DLB and PDD.

In the LBD group, for correlations with grey matter volume, the minimum cluster sizes as estimated by 3dClustSim in AFNI were 251 voxels for mu, 249 voxels for sigma, and 236 voxels for tau. For correlations with white matter volume, the estimated minimum cluster sizes were 314 voxels for mu and 304 voxels for sigma. None of the grey or white matter clusters were large enough to survive this correction. They are, therefore, reported as an exploratory analysis at a voxel-level threshold of *p* < 0.001. When considering clusters with *p* < 0.001 (uncorrected), mu was negatively correlated with grey matter volume in a cluster at the right lingual gyrus and frontal pole and with smaller clusters at the right paracingulate gyrus and thalamus (see Supplementary Table S6). Mu and sigma were both negatively correlated with grey matter volume in the right supplementary motor area. Furthermore, sigma was negatively correlated with bilateral frontal and subcortical regions (left and right thalamus, bilateral basal ganglia, and right amygdala), right temporal pole, and precuneus. Tau was positively correlated with grey matter volume in the bilateral cerebellum and the left frontal pole.

At an uncorrected voxel-level threshold of *p* < 0.001, mu and sigma were both negatively correlated with white matter volume in frontal regions and around the primary motor cortices and supplementary motor areas (see Supplementary Table S6). There were no significant correlations between white matter volume and tau.

### Correlations with grey and white matter volume in Alzheimer’s disease

In the AD group, for correlations with grey matter volume, the minimum cluster sizes as estimated by 3dClustSim in AFNI were 282 voxels for mu, 266 voxels for sigma, and 260 voxels for tau. For correlations with white matter volume, the estimated minimum cluster sizes were 264 voxels for mu, 279 voxels for sigma, and 283 voxels for tau.

**Fig. 3:**
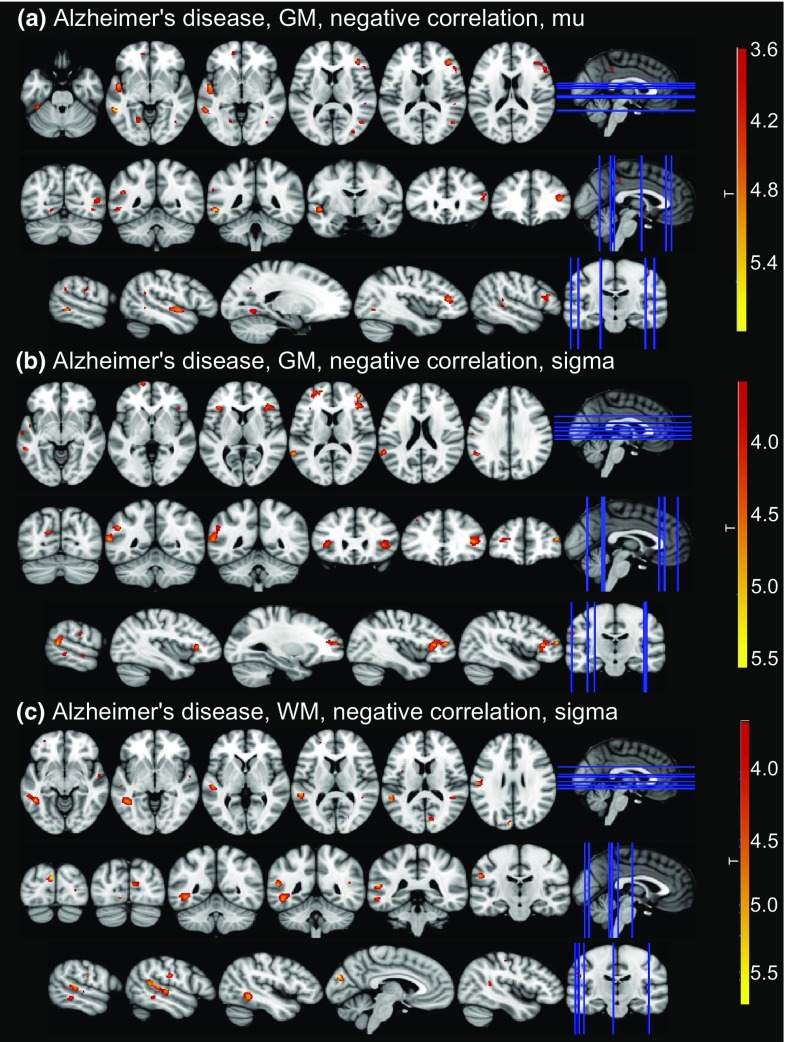
Correlations between ex-Gaussian parameters and grey matter (GM) and white matter (WM) volume for contrasts with clusters that survived multiple comparison correction. Clusters of significant correlations are overlaid on the MNI standard brain in radiological convention, i.e., the right side of the image corresponds to the left hemisphere. See Supplementary Tables S6 and S7 for all uncorrected clusters

At an uncorrected voxel-level threshold of *p* < 0.001, mu was negatively correlated with numerous clusters in widespread parts of the brain including bilateral occipital, frontal, and temporal cortices (see Fig. 3 and Supplementary Table S7). Two larger clusters in right frontal and left temporal regions survived correction for multiple comparisons. Sigma was negatively correlated with grey matter volume in right frontal pole and left supramarginal gyrus (after correction for multiple comparisons), and smaller clusters in left temporal and frontal regions, and the precuneus. There was a positive correlation between sigma and grey matter volume in bilateral temporal gyri and the cerebellum. Tau was negatively correlated with clusters in the right cerebellum.

There were two larger clusters of negative correlation between sigma and white matter volume in left temporal gyrus that survived multiple comparison correction (see Fig. 3 and Supplementary Table S7). At an uncorrected voxel-level threshold of *p* < 0.001, mu was negatively correlated with white matter volume in middle temporal regions and tau was negatively correlated with white matter volume in left lingual regions.

## Discussion

In this study, we investigated changes in reaction time performance in LBD and AD compared to healthy ageing using an ex-Gaussian distributional analysis. We observed differential effects for the different ex-Gaussian parameters indicating that different aspects of reaction time distributions are differentially affected by the two forms of dementia. The two dementia groups could be distinguished by a relative lack of an overall reaction time slowing in the AD group compared to the LBD group. While dementia in general led to more fluctuations in reaction time performance as indicated by an increase in tau, this did not appear to be associated with the clinical fluctuations observed in LBD patients. We found widespread correlations between the Gaussian parameters and grey and white matter volume in AD, whereas there was a relative lack of significant results in the LBD patients with respect to correlations between reaction time performance and cortical volume.

### More extremely slow responses in Alzheimer’s disease compared to controls

Results from a previous standard reaction time analysis in the same group of participants suggested a slowing of reaction time performance in both dementia groups as indicated by an increased mean reaction time with more pronounced deficits in LBD compared to AD [24]. The present ex-Gaussian distributional analysis allows for a more detailed and specific characterization of reaction time performance changes in dementia. The lack of a significant increase in the mean of the Gaussian component in AD compared to controls indicates that there is no major overall slowing of reaction times in our AD group compared to healthy ageing, contrary to what was suggested by the results of the standard reaction time analysis using arithmetic mean reaction times. In contrast, the increased tau parameter shows that the overall increase in mean reaction time in AD patients is being driven by an increase in extremely slow responses. These can be seen as temporary attentional lapses in a subset of trials and are thought to be more directly linked to attentional difficulties than an overall slowing of reaction times [33]. It has also been argued that tau reflects more dynamic processes of attention such as attentional control and working memory; an increase in tau might thus reflect a breakdown of attentional control systems and poorer working memory capacities in AD [14, 15]. The present result is in line with previous studies that have consistently associated mild AD with an increase in tau and no change in mu or sigma across different tasks [13, 14]. The same has been reported in non-demented individuals who later converted to AD, suggesting that an increase in tau might be a very early indicator of the disease [34]. Our results show that the overall pattern observed in preclinical and early stage AD persists in patients at a mild to moderate stage of dementia. However, in addition to an increase in tau we also found a trend for a larger mu in AD compared to controls, indicating that a more general slowing of reaction times might develop in AD patients as the disease progresses. This hypothesis is supported by a negative correlation between mu and dementia severity as measured by the MMSE, suggesting that more severe dementia is related to slower overall reaction times in the AD group.

### Overall reaction time slowing in Lewy body dementia compared to Alzheimer’s disease and controls

This is the first ex-Gaussian study of reaction time distributions in LBD patients. In addition to the increase in excessively slow responses that was observed in both dementia groups, LBD patients also showed an overall slowing (increased mu) and higher variability (increased sigma) compared to controls. In addition, this slowing of reaction times was significantly larger than in the AD group and might be linked to greater attentional impairment in LBD relative to AD [6, 7]. However, we did not observe any difference between the two dementia groups with respect to the slow tail of the distribution (tau), i.e., there was no further increase in attentional lapses in LBD patients compared to AD. This is in contrast to our hypothesis given that tau is thought to capture attentional fluctuations [15] which are a core symptom of LBD and less common in AD [16]. However, the correlation analysis with the clinical fluctuation scales did not reveal any significant correlation between the severity of cognitive fluctuations and any of the ex-Gaussian parameters, suggesting that tau, contrary to our hypothesis, might not be a suitable marker for cognitive fluctuations in LBD. It also suggests that the fluctuations that are commonly observed in LBD patients and that are measured by clinical scales might not correspond well to trial-to-trial fluctuations that are observed upon the execution of the ANT task. This is in contrast to early studies in DLB that found a positive relation between the severity of cognitive fluctuations and trial-to-trial fluctuations on choice reaction time tasks [8–10]. However, LBD patients in earlier studies were more impaired than the present LBD group and in contrast to most of our patients, they were not taking acetylcholinesterase inhibitors [8–10]. Cholinesterase inhibitors have been shown to reduce reaction time variability in patients with cognitive fluctuations [35]. The discrepancy between the present results and previous studies could, therefore, indicate that the association between clinical fluctuation severity and trial-to-trial reaction time variability might be specific to unmedicated LBD patients.

We observed a correlation trend between the Gaussian parameters (mu and sigma) and the severity of Parkinsonism, suggesting that motor problems in LBD might have an influence on the general slowing of reaction time performance [9]. This is supported by the fact that the PDD group which generally showed more severe motor impairment also seemed to be more impaired in terms of the ex-Gaussian parameters than the DLB patients. However, these differences did not remain significant after correcting for multiple comparisons.

### Structural correlates of reaction time deficits in Lewy body dementia and Alzheimer’s disease

This is the first investigation assessing the association between ex-Gaussian parameters and cortical volume in LBD. We found more significant correlations between grey and white matter loss and reaction time deficits in AD than in LBD, indicating that attentional deficits in AD might be more strongly linked to regional brain volume than in LBD.

The AD group showed negative correlations between grey matter volume in widespread cortical regions, such as temporal, lingual, and left frontal cortex, and the Gaussian part of the reaction time distribution (mu and sigma). Furthermore, white matter loss in temporal regions was related to increased sigma which corroborates the grey matter results. In contrast, white matter volume was not significantly related to mu which is in line with previous studies that have suggested that reaction time variability might be more related to white matter changes than mean reaction time [36, 37]. Both mu and sigma also correlated with the MMSE, suggesting that correlations between cortical volume and these ex-Gaussian parameters may be, at least partly, related to global cognition in AD. This is supported by the negative correlations between mu and sigma and grey matter volume at several brain regions related to the default mode network such as the occipital cortex, temporal gyrus, paracingulate cortex, frontal pole, and the precuneus [38]. The default mode network is a brain system associated with memory recall and is highly affected by AD pathology [39].

When considering results at an uncorrected threshold, our LBD group showed negative correlations in the frontal cortex, specifically between the right frontal pole and reaction time variability (sigma). This agrees with Sanchez-Castaneda et al. [40] who reported a relation between a reduction in cortical volume within frontal regions and worse performance on a test of maintained attention and response inhibition in LBD. On the contrary, tau showed a positive correlation with left cerebellar Crus I. This region has been associated with the dorsal attention network [41] and has been found to be structurally altered in DLB [42]. The observed association of a higher number of very slow responses with an increase in grey matter volume might seem counter-intuitive. However, it may represent an imbalance within the attention system, where a structurally intact cerebellum may drive an “over-thinking” in the decision making during the ANT task, causing higher values of tau. Although non-significant after multiple comparison correction these regions showed some correspondence with the results in the AD group. Many LBD cases, especially those with DLB, exhibit significant concurrent AD pathology [43, 44] which has been related to higher global atrophy rates in these patients [45–47] and may explain some of these similarities.

However, the overall lack of significant VBM correlations in the LBD group suggests that attentional dysfunction in LBD might be more related to microstructural changes at the synaptic level that are not observable by volume estimators such as VBM [48]. In line with this hypothesis, there is evidence that Lewy body pathology might play a role in disrupting the structure and function of synapses in LBD [49, 50].

Another possible explanation would be strategic neuronal loss in key widespread cortico-petal networks such as the cholinergic or noradrenergic systems which are profoundly pathological in LBD [51–54], but are difficult to discern in standard structural neuroimaging.

### Limitations

The ANT was originally designed to probe three different aspects of attention by comparing reaction time performance between the different cue and target conditions (alerting effect: no cue—neutral cue, orienting effect: neutral cue-spatial cue, executive conflict effect: incongruent target—congruent target [25]). In the present analysis we combined trials from all conditions and only considered overall effects on the three ex-Gaussian parameters. While the ex-Gaussian analysis provides a useful tool to separate different parts of the reaction time distribution, a problematic aspect is its need for a relatively high number of trials to obtain a good model fit. The low number of trials that was available for each cue and target condition did not allow for a successful fit of the ex-Gaussian distribution to each condition separately. Therefore, it was not feasible to perform the ex-Gaussian analysis for the different components of the ANT. Future studies with a larger number of trials will be needed to study the effect of the different ANT conditions on the three ex-Gaussian parameters.

## Conclusions

This study shows that different aspects of reaction time performance are differentially affected by AD and LBD. Furthermore, we showed that the neural correlates of impaired attentional performance differ between the two forms of dementia. While impaired reaction time performance is linked to grey and white matter atrophy in AD, the more pronounced behavioural deficits that we observed in the LBD group did not exhibit strong correlations with brain structure. They, therefore, seem to be a functional or microstructural rather than a macrostructural phenomenon. However, future work using functional MRI or diffusion tensor imaging will be required to further elucidate this.

## Electronic supplementary material

Below is the link to the electronic supplementary material.

Supplementary material 1 (DOCX 547 kb)

## References

[1] Vann Jones SA, O’Brien JT (2014). The prevalence and incidence of dementia with Lewy bodies: a systematic review of population and clinical studies. Psychol Med.

[2] McKeith IG, Dickson DW, Lowe J (2005). Diagnosis and management of dementia with Lewy bodies: third report of the DLB consortium. Neurology.

[3] Aarsland D, Londos E, Ballard CG (2009). Parkinson’s disease dementia and dementia with Lewy bodies: different aspects of one entity. Int Psychogeriatr.

[4] McKeith IG (2007). Dementia with Lewy bodies and Parkinson’s disease with dementia: where two worlds collide. Pract Neurol.

[5] Metzler-Baddeley C (2007). A review of cognitive impairments in dementia with Lewy bodies relative to Alzheimer’s disease and Parkinson’s disease with dementia. Cortex.

[6] Ballard CG, O’Brien JT, Gray A (2001). Attention and fluctuating attention in patients with dementia with Lewy bodies and Alzheimer disease. Arch Neurol.

[7] Bradshaw JM, Saling M, Anderson V (2006). Higher cortical deficits influence attentional processing in dementia with Lewy bodies, relative to patients with dementia of the Alzheimer’s type and controls. J Neurol Neurosurg Psychiatry.

[8] Ballard CG, Walker M, O’Brien JT (2001). The characterisation and impact of “fluctuating” cognition in dementia with Lewy bodies and Alzheimer’s disease. Int J Geriatr Psychiatry.

[9] Ballard CG, Aarsland D, McKeith IG (2002). Fluctuations in attention: PD dementia vs DLB with parkinsonism. Neurology.

[10] Walker MP, Ayre GA, Cummings JL (2000). Quantifying fluctuation in dementia with Lewy bodies, Alzheimer’s disease, and vascular dementia. Neurology.

[11] Balota DA, Yap MJ (2011). Moving beyond the mean in studies of mental chronometry: the power of response time distributional analyses. Curr Dir Psychol Sci.

[12] Ratcliff R (1979). Group reaction time distributions and an analysis of distribution statistics. Psychol Bull.

[13] Jackson JD, Balota DA, Duchek JM, Head D (2012). White matter integrity and reaction time intraindividual variability in healthy aging and early-stage Alzheimer disease. Neuropsychologia.

[14] Tse CS, Balota DA, Yap MJ (2010). Effects of healthy aging and early-stage dementia of the Alzheimer’s type on components of response time distributions in three attention tasks. Neuropsychology.

[15] Schmiedek F, Oberauer K, Wilhelm O (2007). Individual differences in components of reaction time distributions and their relations to working memory and intelligence. J Exp Psychol Gen.

[16] Bradshaw J, Saling M, Hopwood M (2004). Fluctuating cognition in dementia with Lewy bodies and Alzheimer’s disease is qualitatively distinct. J Neurol Neurosurg Psychiatry.

[17] Bonanni L, Perfetti B, Bifolchetti S (2015). Quantitative electroencephalogram utility in predicting conversion of mild cognitive impairment to dementia with Lewy bodies. Neurobiol Aging.

[18] Lee DR, Taylor JP, Thomas AJ (2012). Assessment of cognitive fluctuation in dementia: a systematic review of the literature. Int J Geriatr Psychiatry.

[19] Emre M, Aarsland D, Brown R (2007). Clinical diagnostic criteria for dementia associated with Parkinson’s disease. Mov Disord.

[20] McKhann GM, Knopman DS, Chertkow H (2011). The diagnosis of dementia due to Alzheimer’s disease: recommendations from the National Institute on Aging-Alzheimer’s Association workgroups on diagnostic guidelines for Alzheimer’s disease. Alzheimer’s Dement.

[21] Walker Z, Costa DC, Walker RWH (2002). Differentiation of dementia with Lewy bodies from Alzheimer’s disease using a dopaminergic presynaptic ligand. J Neurol Neurosurg Psychiatry.

[22] McKeith IG, O’Brien JT, Walker Z (2007). Sensitivity and specificity of dopamine transporter imaging with ^123^I-FP-CIT SPECT in dementia with Lewy bodies: a phase III, multicentre study. Lancet Neurol.

[23] Firbank MJ, Kobeleva X, Cherry G (2016). Neural correlates of attention-executive dysfunction in Lewy body dementia and Alzheimer’s disease. Hum Brain Mapp.

[24] Cromarty RA, Schumacher J, Graziadio S (2018). Structural brain correlates of attention dysfunction in Lewy body dementias and Alzheimer’s disease. Front Aging Neurosci.

[25] Fan J, McCandliss BD, Sommer T (2002). Testing the efficiency and independence of attentional networks. J Cogn Neurosci.

[26] Hoogendam YY, Hofman A, van der Geest JN (2014). Patterns of cognitive function in aging: the Rotterdam Study. Eur J Epidemiol.

[27] Eriksen BA, Eriksen CW (1974). Effects of noise letters upon the identification of a target letter in a nonsearch task. Percept Psychophys.

[28] Posner MI, Petersen SE (1990). The attention system of the human brain. Annu Rev Neurosci.

[29] Lacouture Y, Cousineau D (2008). How to use MATLAB to fit the ex-Gaussian and other probability functions to a distribution of response times. Tutor Quant Methods Psychol.

[30] Ashburner J (2007). A fast diffeomorphic image registration algorithm. Neuroimage.

[31] Ridgway GR, Omar R, Ourselin S (2009). Issues with threshold masking in voxel-based morphometry of atrophied brains. Neuroimage.

[32] Burton EJ, McKeith IG, Burn DJ (2004). Cerebral atrophy in Parkinson’s disease with and without dementia: a comparison with Alzheimer’s disease, dementia with Lewy bodies and controls. Brain.

[33] Hervey AS, Epstein JN, Curry JF (2006). Reaction time distribution analysis of neuropsychological performance in an ADHD sample. Child Neuropsychol.

[34] Balota DA, Tse CS, Hutchison KA (2010). Predicting conversion to dementia of the Alzheimer’s type in a healthy control sample: the power of errors in Stroop color naming. Psychol Aging.

[35] Onofrj M, Thomas A, Iacono D (2003). The effects of a cholinesterase inhibitor are prominent in patients with fluctuating cognition: a part 3 study of the main mechanism of cholinesterase inhibitors in dementia. Clin Neuropharmacol.

[36] Moy G, Millet P, Haller S (2011). Magnetic resonance imaging determinants of intraindividual variability in the elderly: combined analysis of grey and white matter. Neuroscience.

[37] Mella N, de Ribaupierre S, Eagleson R, de Ribaupierre A (2013). Cognitive intraindividual variability and white matter integrity in aging. Sci World J.

[38] Mevel K, Chételat G, Eustache F, Desgranges B (2011). The default mode network in healthy aging and Alzheimer’s disease. Int J Alzheimers Dis.

[39] Agosta F, Pievani M, Geroldi C (2012). Resting state fMRI in Alzheimer’s disease: beyond the default mode network. Neurobiol Aging.

[40] Sanchez-Castaneda C, Rene R, Ramirez-Ruiz B (2009). Correlations between gray matter reductions and cognitive deficits in dementia with Lewy Bodies and Parkinson’s disease with dementia. Mov Disord.

[41] Peraza LR, Kaiser M, Firbank MJ (2014). fMRI resting state networks and their association with cognitive fluctuations in dementia with Lewy bodies. NeuroImage Clin.

[42] Colloby SJ, O’Brien JT, Taylor JP (2014). Patterns of cerebellar volume loss in dementia with Lewy bodies and Alzheimer’s disease: a VBM-DARTEL study. Psychiatry Res Neuroimaging.

[43] Dugger BN, Adler CH, Shill HA (2014). Concomitant pathologies among a spectrum of parkinsonian disorders. Parkinsonism Relat Disord.

[44] Howlett DR, Whitfield D, Johnson M (2015). Regional multiple pathology scores are associated with cognitive decline in Lewy body dementias. Brain Pathol.

[45] Nedelska Z, Ferman TJ, Boeve BF (2015). Pattern of brain atrophy rates in autopsy-confirmed dementia with Lewy bodies. Neurobiol Aging.

[46] Sarro L, Senjem ML, Lundt ES (2016). Amyloid-β deposition and regional grey matter atrophy rates in dementia with Lewy bodies. Brain.

[47] Shimada H, Shinotoh H, Hirano S (2013). β-Amyloid in Lewy body disease is related to Alzheimer’s disease-like atrophy. Mov Disord.

[48] Kramer ML, Schulz-Schaeffer WJ (2007). Presynaptic alpha-synuclein aggregates, not Lewy bodies, cause neurodegeneration in dementia with Lewy bodies. J Neurosci.

[49] Colom-Cadena M, Pegueroles J, Herrmann AG (2017). Synaptic phosphorylated α-synuclein in dementia with Lewy bodies. Brain.

[50] Calo L, Wegrzynowicz M, Santivañez-Perez J, Grazia Spillantini M (2016). Synaptic failure and α-synuclein. Mov Disord.

[51] Lippa CF, Smith TW, Perry E (1999). Dementia with Lewy bodies: choline acetyltransferase parallels nucleus basalis pathology. J Neural Transm.

[52] Shimada H, Hirano S, Shinotoh H (2009). Mapping of brain acetylcholinesterase alterations in Lewy body disease by PET. Neurology.

[53] Colloby SJ, Elder GJ, Rabee R (2017). Structural grey matter changes in the substantia innominata in Alzheimer’s disease and dementia with Lewy bodies: a DARTEL-VBM study. Int J Geriatr Psychiatry.

[54] Del Tredici K, Braak H (2013). Dysfunction of the locus coeruleus-norepinephrine system and related circuitry in Parkinson’s disease-related dementia. J Neurol Neurosurg Psychiatry.

