# Supplementary material for: Structural correlates of attention dysfunction in Lewy body dementia and Alzheimer’s disease: an ex-Gaussian analysis
Source: J Neurol. 2019 Apr 21;266(7):1716–26. doi: 10.1007/s00415-019-09323-y (PMC6586700; doi:10.1007/s00415-019-09323-y)
Supplement: Supplementary file 1 — Supplementary material 1 (DOCX 547 kb) [file 415_2019_9323_MOESM1_ESM.docx]

Supplementary Material: Structural correlates of attention dysfunction in Lewy body dementia and Alzheimer’s disease: An ex-Gaussian analysis

Julia Schumacher*, Ruth Cromarty, Peter Gallagher, Michael J. Firbank, Alan J. Thomas, Marcus Kaiser, Andrew M. Blamire, John T. O’Brien, Luis R. Peraza, John-Paul Taylor

* Correspondence: j.a.schumacher2@newcastle.ac.uk

Contents

1. Comparison of demographics and clinical variables in DLB and PDD subgroups
2. Comparison of demographics and clinical variables for matched AD and LBD subgroups
3. Comparison of ex-Gaussian parameters for matched dementia groups
4. Correlations with cognitive fluctuation scores in the LBD group
5. Correlations between grey and white matter volume and ex-Gaussian parameters
6. VBM analysis of group differences in grey matter volume

1. Comparison of demographics and clinical variables in DLB and PDD subgroups

Table S1: Demographic and clinical comparison of DLB and PDD patients

|  | DLB (N=23) | PDD (N=16) | Between-group differences |
| --- | --- | --- | --- |
| Male: female | 18:5 | 16:0 | χ^2^=3.99, p=0.046^a^ |
| Age | 76.4 (5.9) | 74.2 (4.7) | t_37_=1.27, p=0.21^c^ |
| AChEI | 21 | 14 | χ^2^ =0.15, p =0.70^a^ |
| PD meds | 12 | 16 | χ^2^ =10.66, p=0.001^a^ |
| Duration | 3.4 (2.1) | 2.6 (1.5) | U =142.50, p=0.22^b^ |
| MMSE | 23.5 (4.0) | 23.4 (3.4) | t_37_ =0.12, p =0.91^c^ |
| CAMCOG | 76.0 (14.4) | 76.5 (9.6) | t_37_ =0.13, p =0.90^c^ |
| UPDRS | 14.3 (5.3) | 26.2 (7.0) | t_37_ =6.08, p<0.001^c^ |
| CAF total | 3.8 (4.5) | 6.6 (4.6) | t_37_ =1.93, p=0.06^c^ |
| Mayo total | 11.9 (6.1) | 15.0 (5.3) | t_37_=1.65, p=0.11^c^ |
| Mayo cogn | 2.1 (1.8) | 3.5 (1.8) | t_37_=2.35, p=0.02^c^ |
| NPI total | 8.7 (4.8) | 20.1 (12.5) | t_37_=3.99, p<0.001^c^ |
| NPI hall | 1.4 (1.7) | 2.1 (2.5) | t_37_=1.09, p=0.28^c^ |

AChEI, number of patients taking acetylcholinesterase inhibitors; CAF total, Clinical Assessment of Fluctuations total score; CAMCOG, Cambridge Cognitive Examination; DLB, Dementia with Lewy bodies; Duration, duration of cognitive symptoms in years; Mayo total, Mayo Fluctuations Scale; Mayo cognitive, Mayo Fluctuation cognitive subscale; Mayo arousal, Mayo Fluctuations arousal subscale; MMSE, Mini Mental State Examination; PDD, Parkinson’s disease dementia; PD meds, number of patients taking dopaminergic medication; UPDRS, Unified Parkinson’s Disease Rating Scale; NPI, Neuropsychiatric Inventory; NPI hall, NPI hallucination subscore
^a^ Chi-square test DLB, PDD; ^b^ Mann Whitney U test DLB, PDD; ^c^ Student’s t-test DLB, PDD.

2. Comparison of demographics and clinical variables for matched AD and LBD subgroups


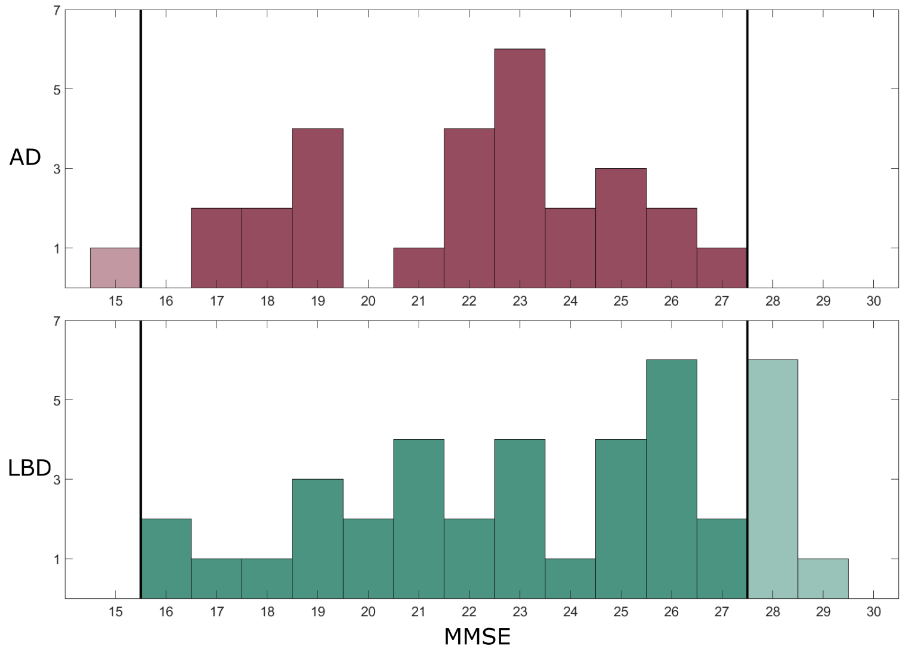


Figure S1: Selection of matched dementia groups by excluding one Alzheimer’s disease patient with MMSE<16 and seven Lewy body dementia (five dementia with Lewy bodies and two Parkinson’s disease dementia) patients with MMSE>27.

Table S2: Demographics and clinical information for matched AD and LBD subgroups, mean (standard deviation)

|  | HC (N=22) | AD (N=27) | LBD (N=32) | Between-group differences |
| --- | --- | --- | --- | --- |
| Male: female | 15:7 | 22:5 | 28:4 | χ^2^=3.11, p=0.21^a^ |
| Age | 75.9 (5.4) | 76.2 (7.9) | 75.6 (5.6) | F_2,78_=0.06, p=0.95^b^ |
| AChEI | na | 26 | 30 | χ^2^=0.20, p=0.66^c^ |
| PD meds | na | 0 | 23 | χ^2^=31.81, p<0.001^c^ |
| Duration | na | 3.8 (2.1) | 3.1 (1.9) | U=329, p=0.11^d^ |
| MMSE | 29.2 (0.9) | 22.0 (2.9) | 22.4 (3.3) | t_57_=0.49, p=0.63^e^ |
| CAMCOG | 96.7 (3.7) | 72.1 (10.1) | 72.9 (11.3) | t_57_=0.31, p=0.76^e^ |
| UPDRS | 1.1 (1.4) | 2.1 (2.0) | 19.3 (8.6) | t_57_=10.17, p<0.001^e^ |
| CAF total | na | 0.8 (1.7)^f^ | 5.3 (4.9) | t_56_=4.47, p<0.001^e^ |
| Mayo total | na | 8.7 (4.0)^f^ | 14.3 (5.4) | t_56_=4.40, p<0.001^e^ |
| Mayo cogn | na | 1.8 (1.8)^f^ | 3.0 (1.8) | t_56_=2.54, p=0.01^e^ |
| NPI total | na | 6.9 (6.5)^f^ | 14.1 (10.5) | t_56_=3.06, p=0.003^e^ |
| NPI hall | na | 0.04 (0.2)^f^ | 1.5 (1.8) | t_56_=4.09, p<0.001^e^ |

AChEI, number of patients taking acetylcholinesterase inhibitors; AD, Alzheimer’s disease; CAF total, Clinical Assessment of Fluctuations total score; CAMCOG, Cambridge Cognitive Examination; Duration, duration of cognitive symptoms in years; HC, healthy controls; LBD, Lewy body dementia; Mayo cogn, Mayo Fluctuations cognitive subscale; Mayo total, Mayo Fluctuations Scale; Mayo arousal, Mayo Fluctuations arousal subscale; MMSE, Mini Mental State Examination; na, not applicable; PD meds, number of patients taking dopaminergic medication; UPDRS, Unified Parkinson’s Disease Rating Scale III; NPI, Neuropsychiatric Inventory; NPI hall, NPI hallucination subscore
^a^ Chi-square test HC, AD, DLB; ^b^ One-way ANOVA HC, AD, DLB; ^c^ Chi-square test AD, DLB; ^d^ Mann Whitney U test AD, DLB; ^e^ Student’s t-test AD, DLB.

^f^ N=26

3. Comparison of ex-Gaussian parameters for matched dementia groups

Table S3: Ex-Gaussian parameters, mean (standard deviation) for matched dementia subgroups (see Supplementary Figure S1 and Supplementary Table S2). Between-group differences were assessed by Kruskal-Wallis tests with Dunn’s post-hoc tests, Bonferroni-corrected for multiple comparisons.

|  | HC | AD | LBD | Kruskal-Wallis | post-hoc tests | | |
| --- | --- | --- | --- | --- | --- | --- | --- |
|  |  |  |  |  | HC vs AD | HC vs LBD | AD vs LBD |
| Mu | 649.43 (73.88) | 738.21 (129.81) | 958.61 (162.50) | F_2_=42.57, p<0.001 | p=0.10 | p<0.001 | p<0.001 |
| Sigma | 59.85 (22.87) | 76.34 (46.34) | 127.80 (65.09) | F_2_=22.55, p<0.001 | p=1.0 | p<0.001 | p=0.001 |
| Tau | 313.48 (119.50) | 522.70 (206.74) | 616.30 (204.76) | F_2_=27.62, p<0.001 | p=0.001 | p<0.001 | p=0.36 |

AD, Alzheimer’s disease; HC, healthy controls: LBD, Lewy body dementia

Table S4: Mean (standard deviation), Kruskal-Wallis test for between-group differences with post-hoc Dunn's test (Bonferroni corrected for multiple comparisons), treating DLB and PDD as separate groups.

|  | HC | AD | DLB | PDD | Kruskal-Wallis | post-hoc tests | | | | | |
| --- | --- | --- | --- | --- | --- | --- | --- | --- | --- | --- | --- |
|  |  |  |  |  |  | HC vs AD | HC vs DLB | HC vs PDD | AD vs DLB | AD vs PDD | DLB vs PDD |
| Mu | 649.43 (73.88) | 748.90 (139.38) | 879.89 (143.68) | 1003.81 (173.36) | F_3_=42.77, p<0.001 | p=0.11 | p<0.001 | p<0.001 | p=0.039 | p=0.001 | p=0.79 |
|  |  |  |  |  |  |  |  |  |  |  |  |
| Sigma | 59.85 (22.87) | 78.57 (46.98) | 104.92 (46.69) | 153.94 (72.57) | F_3_=26.08, p<0.001 | p=1.0 | p=0.009 | p<0.001 | p=0.128 | p=0.001 | p=0.554 |
|  |  |  |  |  |  |  |  |  |  |  |  |
| Tau | 313.48 (119.50) | 523.28 (202.89) | 545.86 (226.29) | 611.99 (193.15) | F_3_=25.29, p<0.001 | p=0.001 | p=0.001 | p<0.001 | p=1.0 | p=1.0 | p=1.0 |

AD, Alzheimer’s disease; DLB, dementia with Lewy bodies; HC, healthy controls; PDD, Parkinson’s disease dementia

4. Correlations with cognitive fluctuation scores in the LBD group

Table S5: Spearman’s rank correlations of Ex-Gaussian parameters and overall performance with clinical fluctuations scores in LBD, correlation coefficient (p-value, uncorrected)

|  | LBD | | |  |  |
| --- | --- | --- | --- | --- | --- |
|  | Mayo total | Mayo arousal | CAF total | CAF duration | CAF frequency |
| Mu | 0.23 (0.15) | 0.07 (0.67) | 0.09 (0.60) | 0.03 (0.86) | 0.12 (0.45) |
| Sigma | 0.20 (0.22) | 0.03 (0.86) | 0.12 (0.49) | 0.08 (0.63) | 0.17 (0.29) |
| Tau | 0.26 (0.11) | 0.07 (0.66) | 0.04 (0.83) | 0.05 (0.74) | 0.03 (0.85) |
| % correct | -0.13 (0.43) | -0.003 (0.98) | -0.13 (0.43) | -0.19 (0.26) | -0.004 (0.98) |

CAF, Clinician Assessment of Fluctuation; Mayo, Mayo Fluctuations scale; % correct, percentage of correct trials across all included runs.

5. Correlations between grey and white matter volume and ex-Gaussian parameters

Table S6: Correlations between ex-Gaussian parameters and grey matter (GM) and white matter (WM) volume from VBM analysis in LBD. All clusters are significant at an uncorrected p<0.001. Correction for multiple comparisons was performed using 3dClustSim in AFNI at p<0.05 which resulted in a minimum cluster size of 251 voxels for mu (GM), 314 voxels for mu (WM), 249 voxels for sigma (GM), 304 voxels for sigma (WM), and 236 voxels for tau (GM). No clusters survived multiple comparison correction. Anatomical labels were determined from the Harvard-Oxford Structural Atlas.

| **Grey matter** | | | **White matter** | | |
| --- | --- | --- | --- | --- | --- |
| Cluster location | size | MNI (X,Y,Z) | Cluster location | size | MNI (X,Y,Z) |
| **Mu, negative correlation** | | | | | |
| R lingual gyrus | 63 | 6, -92, -14 | L precentral gyrus | 186 | -16,-27,57 |
| R white matter | 37 | 12, 30, 45 | R superior frontal gyrus | 26 | 14,4,63 |
| R frontal pole | 34 | 12, 54, 34 | R superior frontal gyrus | 26 | 22, 24, 40 |
| R thalamus | 20 | 3, -16, 10 | R superior frontal gyrus | 10 | 9, 34, 44 |
| R paracingulate gyrus | 16 | 3, 42, 34 | R frontal pole | 9 | 15, 48, 34 |
| R supplementary motor area | 14 | 12, 0, 54 | R supplementary motor area | 7 | 10, -3, 48 |
| R thalamus | 6 | 18, -34, 8 | L cerebellum VIIb | 6 | -24,-69,-45 |
| L thalamus | 5 | -20, -34, 6 | R frontal pole | 1 | 42, 36, 12 |
| R middle frontal gyrus | 1 | 38, 28, 22 | R inferior frontal gyrus | 1 | 50, 12, 16 |
| **Mu, positive correlation** | | | | | |
| No significant clusters | | | No significant clusters |  |  |
| **Sigma, negative correlation** | | | | | |
| R frontal pole | 169 | 33, 40, 26 | L frontal pole | 146 | -22, 51,-14 |
| L middle frontal gyrus | 152 | -39, 14, 45 | L frontal pole | 71 | -34, 46, -9 |
| R superior frontal gyrus | 116 | 14, 9, 56 | R frontal pole | 32 | 44, 42, -8 |
| L inferior frontal gyrus | 111 | -50, 15, 24 | L frontal pole | 24 | -38, 45, 4 |
| L frontal pole | 91 | -27, 39,-10 | R supplementary motor area | 19 | 8, -4, 58 |
| R amygdala | 32 | 22, -8, -9 | L middle frontal gyrus | 14 | -34, 12, 28 |
| R supplementary motor area | 31 | 3, -4, 62 | L frontal pole | 8 | -42,40,-10 |
| R temporal pole | 29 | 26, 6, -21 | L middle frontal gyrus | 7 | -36, 8, 42 |
| R precuneus | 25 | 14, -48, 52 | R superior frontal gyrus | 6 | 9, 46, 33 |
| R middle frontal gyrus | 24 | 34, 12, 33 | L precentral gyrus | 5 | -38, -16,63 |
| R superior frontal gyrus | 18 | 4, 33, 46 |  |  |  |
| R thalamus | 16 | 12, -36, 4 |  |  |  |
| L middle frontal gyrus | 14 | -33, 14, 57 |  |  |  |
| L thalamus | 12 | -10, -36, 3 |  |  |  |
| R caudate | 10 | 8, 18, 0 |  |  |  |
| L pallidum | 9 | -20, -8, -9 |  |  |  |
| L white matter | 9 | -40, 18, 14 |  |  |  |
| R precentral gyrus | 4 | 51, 2, 18 |  |  |  |
| R frontal orbital cortex | 3 | 45, 21, -9 |  |  |  |
| L precentral gyrus | 3 | -36, 2, 30 |  |  |  |
| L white matter | 2 | -34, 22, 28 |  |  |  |
| R white matter | 1 | 52, -44, 21 |  |  |  |
| **Sigma, positive correlation** | | | | | |
| R frontal pole | 12 | 36, 42, 39 | No significant clusters |  |  |
| **Tau, negative correlation** | | | | | |
| No significant clusters |  |  | No significant clusters | | |
| **Tau, positive correlation** | | | | | |
| L cerebellum Crus I | 141 | -39,-70,-34 | No significant clusters |  |  |
| R cerebellum IX | 89 | 12,-58,-39 |  |  |  |
| L frontal pole | 64 | -24,57,27 |  |  |  |
| R cerebellum IX | 62 | 6,-46,-54 |  |  |  |

Table S7: Correlations between ex-Gaussian parameters and grey matter (GM) and white matter (WM) volume from VBM analysis in AD. All clusters are significant at an uncorrected p<0.001. Correction for multiple comparisons was performed using 3dClustSim in AFNI at p<0.05 which resulted in a minimum cluster size of 282 voxels for mu (GM), 264 voxels for mu (WM), 266 voxels for sigma (GM), 279 voxels for sigma (WM), 260 voxels for tau (GM), and 283 voxels for tau (WM). Clusters surviving multiple comparison correction are highlighted with an asterisk. Anatomical labels were determined from the Harvard-Oxford Structural Atlas.

| **Grey matter** | | | **White matter** | | |
| --- | --- | --- | --- | --- | --- |
| Cluster location | size | MNI (X,Y,Z) | Cluster location | size | MNI (X,Y,Z) |
| **Mu, negative correlation** | | | | | |
| R frontal pole | 422* | 42, 36, 14 | L middle temporal gyrus | 91 | -54,-38,-10 |
| L planum polare | 316* | -48, -4, -4 | R middle temporal gyrus | 80 | 42, -48, 9 |
| L middle temporal gyrus | 156 | -54, -45, -6 | L occipital fusiform gyrus | 30 | -32, -69, 0 |
| L lingual gyrus | 127 | -18, -62, -6 |  |  |  |
| R inferior lateral occipital cortex | 90 | 48, -66, 12 |  |  |  |
| L postcentral gyrus | 71 | -56, -15,32 |  |  |  |
| L posterior temporal fusiform cortex | 61 | -40,-40,-30 |  |  |  |
| L paracingulate cortex | 52 | -14, 50,-3 |  |  |  |
| R inferior lateral occipital cortex | 52 | 33, -81, 10 |  |  |  |
| L angular gyrus | 37 | -46, -50,22 |  |  |  |
| R inferior lateral occipital cortex | 34 | 40, -66,-4 |  |  |  |
| R occipital pole | 33 | 34, -94, 6 |  |  |  |
| L posterior cingulate gyrus | 32 | -2, -38, 42 |  |  |  |
| R frontal pole | 29 | 27, 52, -20 |  |  |  |
| R posterior middle temporal gyrus | 21 | 54, -34, 0 |  |  |  |
| R white matter | 19 | 52, -34, 12 |  |  |  |
| L posterior supramarginal gyrus | 17 | -58, -45,27 |  |  |  |
| R middle temporal gyrus | 14 | 46, -57, -2 |  |  |  |
| L angular gyrus | 10 | -54, -57,36 |  |  |  |
| L precentral gyrus | 7 | -62, -3, 6 |  |  |  |
| R white matter | 6 | 14, -78, 22 |  |  |  |
| R cerebellum IX | 6 | 9, -46, -38 |  |  |  |
| L frontal pole | 5 | -18, 57, 28 |  |  |  |
| L cerebellum IX | 4 | -6, -46, -38 |  |  |  |
| R cerebellum Crus I | 4 | 30, -81, 20 |  |  |  |
| L white matter | 3 | -20, -64,30 |  |  |  |
| L temporal occipital fusiform cortex | 1 | -46,-58,-20 |  |  |  |
| **Mu, positive correlation** | | | | | |
| L anterior middle temporal gyrus | 2 | -66, -6, -18 | No significant clusters |  |  |
| **Sigma, negative correlation** | | | | | |
| R frontal pole | 689* | 42, 48, 12 | L superior temporal gyrus | 588* | -54,-38, 12 |
| L posterior supramarginal gyrus | 428* | -60, -50, 12 | L inferior temporal gyrus | 584* | -45, -48,-8 |
| L frontal pole | 245 | -18, 52, 12 | L postcentral gyrus | 144 | -56,-18, 28 |
| L frontal operculum cortex | 178 | -39, 28, 6 | L cuneal cortex | 125 | -4, -87, 26 |
| L middle temporal gyrus | 97 | -54, -44, -8 | R intracalcarine cortex | 99 | 9, -80, 15 |
| L precuneus | 75 | -15,-70, 24 | R supramarginal gyrus | 77 | 45, -44, 15 |
| L frontal pole | 65 | -15, 70, -3 | R superior temporal gyrus | 62 | 56, -6, -9 |
| L postcentral gyrus | 40 | -58,-18, 28 | L middle frontal gyrus | 40 | -28, 6, 42 |
| L planum polare | 30 | -48, -2, -9 | L central opercular cortex | 31 | -34, 2, 15 |
| L precentral gyrus | 17 | -46, 9, 30 | L middle frontal gyrus | 25 | -27, 28, 34 |
| L posterior middle temporal gyrus | 17 | -60, -15, -9 | R postcentral gyrus | 19 | 42, -21, 51 |
| L frontal pole | 10 | -16, 62, 24 | R lateral occipital cortex | 15 | 28, -88, 6 |
| L posterior middle temporal gyrus | 10 | -52, -32, -14 | L lingual gyrus | 13 | -12, -78, -8 |
| L middle frontal gyrus | 7 | -34, 33, 44 | L frontal pole | 11 | -24, 51, 10 |
| L inferior frontal gyrus | 4 | -54, 10, 20 | L frontal pole | 10 | -32, 52,-10 |
| L posterior superior temporal gyrus | 3 | -50, -33, 2 | R precentral gyrus | 6 | 38, -2, 44 |
| L frontal pole | 2 | -44, 46, 15 | L frontal pole | 5 | -36, 46, 6 |
| L white matter | 1 | -50, -18, -3 | R intracalcarine cortex | 5 | 21, -75, 10 |
| L frontal pole | 1 | -38, 45, 28 | L frontal pole | 4 | -33, 38, 24 |
| L frontal pole | 1 | -18, 48, 40 | L occipital fusiform gyrus | 1 | -28, -62, -8 |
| **Sigma, positive correlation** | | | | | |
| R middle temporal gyrus | 28 | 64, 0, -24 | No significant clusters |  |  |
| L anterior middle temporal gyrus | 21 | -66, -6,-16 |  |  |  |
| L cerebellum Crus II | 6 | -33,-86,-45 |  |  |  |
| R posterior inferior temporal gyrus | 3 | 56, -38,-30 |  |  |  |
| R cerebellum Crus II | 2 | 21,-82,-54 |  |  |  |
| R cerebellum Crus II | 1 | 32, -81,-54 |  |  |  |
|  |  |  |  |  |  |
| **Tau, negative correlation** | | | | | |
| R cerebellum Crus I | 143 | 18, -84,-24 | L lingual gyrus | 30 | -10, -72, -6 |
| L temporal occipital fusiform cortex | 34 | -28, -62,-9 | L lingual gyrus | 4 | -21, -63, -8 |
| R superior lateral occipital cortex | 2 | 24, -57, 56 |  | | |
| **Tau, positive correlation** | | | | | |
| R posterior superior temporal gyrus | 9 | 69, -18, 0 | No significant clusters |  |  |
| R frontal pole | 6 | 33, 34, -21 |  |  |  |

6. VBM analysis of group differences in grey matter volume

To assess differences in grey matter (GM) volume between the groups, a VBM analysis was conducted in SPM12 with age, gender, and total intracranial volume as covariates.
The AD group showed clusters of reduced GM volume compared to controls, mainly around the bilateral hippocampi (see Supplementary Figure S1). There were no regions with increased GM volume in AD compared to controls.
The LBD group showed GM loss compared to controls around the fusiform gyrus in both hemispheres, right postcentral gyrus, right parietal areas, and the right cerebellum (see Supplementary Figure S2). Again, there were no regions with increased GM volume compared to controls.
When comparing AD and LBD, the AD group had reduced GM volume in right parahippocampal areas (see Supplementary Figure S3) with no areas showing increased GM volume in AD compared to LBD.
A comparison of DLB and PDD subgroups revealed no significant differences in GM volume.


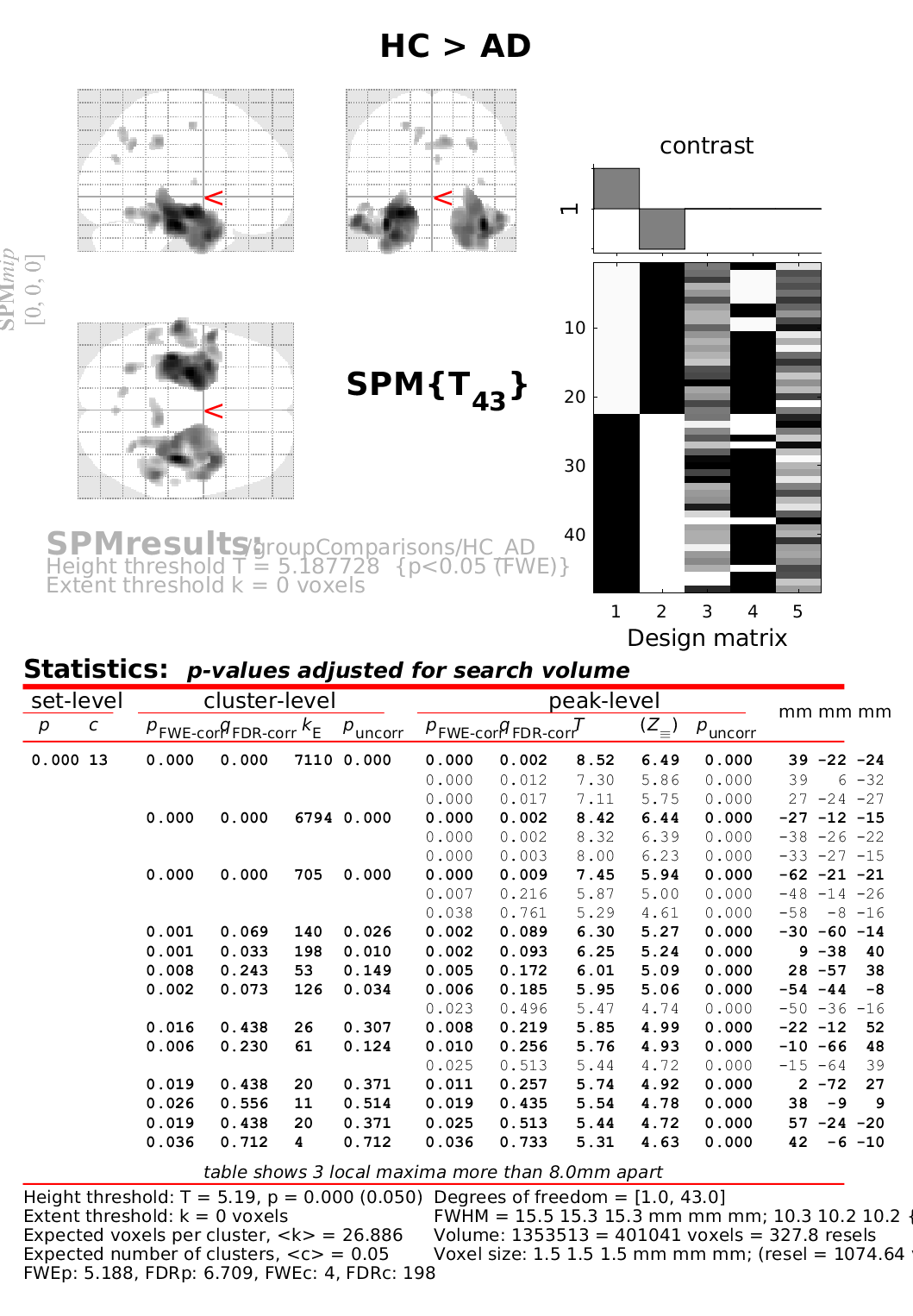


Figure S2: Decrease in GM volume in AD compared to controls, significant clusters are shown for p<0.05 (FWE corrected).


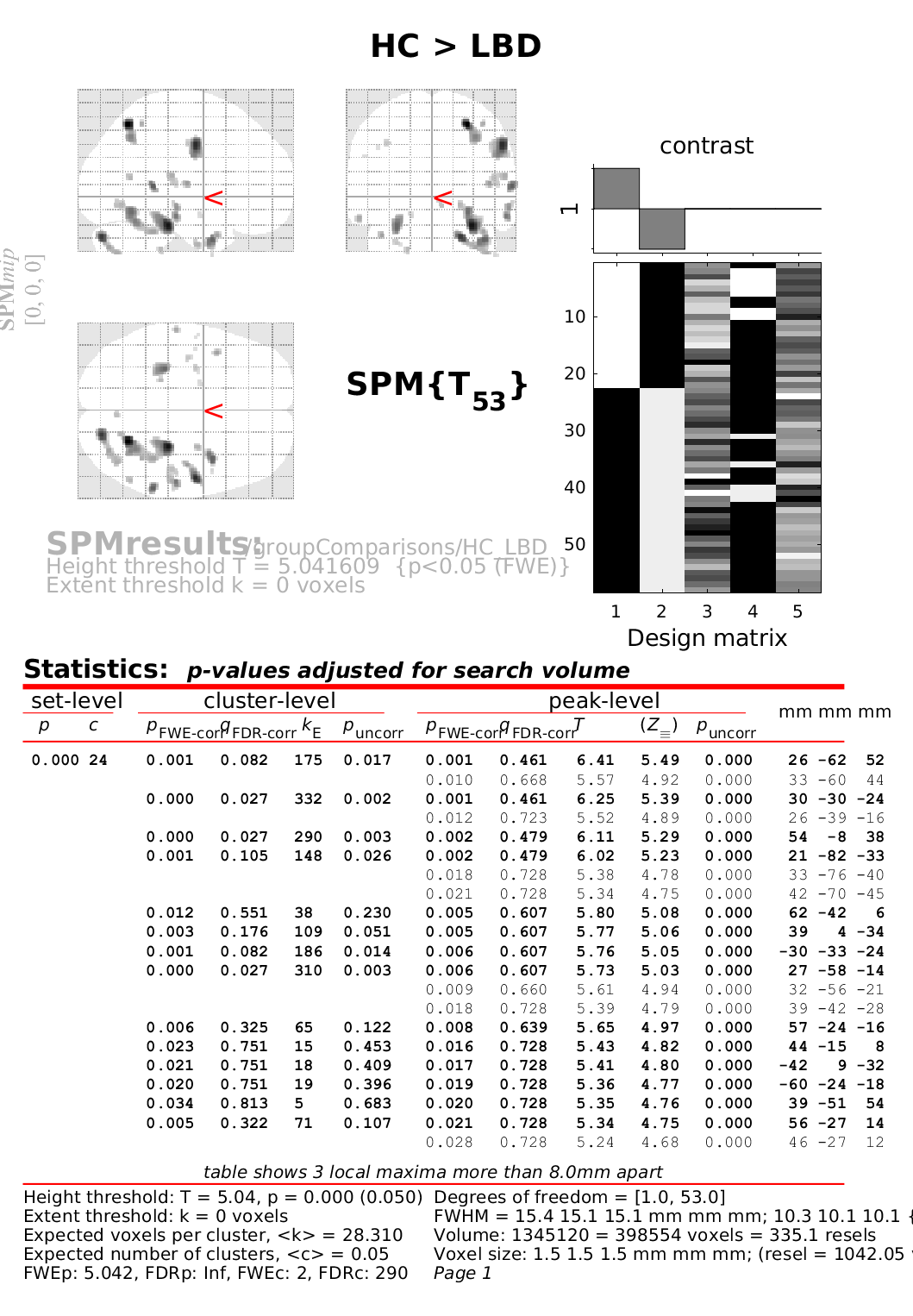


Figure S3: Decrease in GM volume in LBD compared to controls, significant clusters are shown for p<0.05 (FWE corrected).


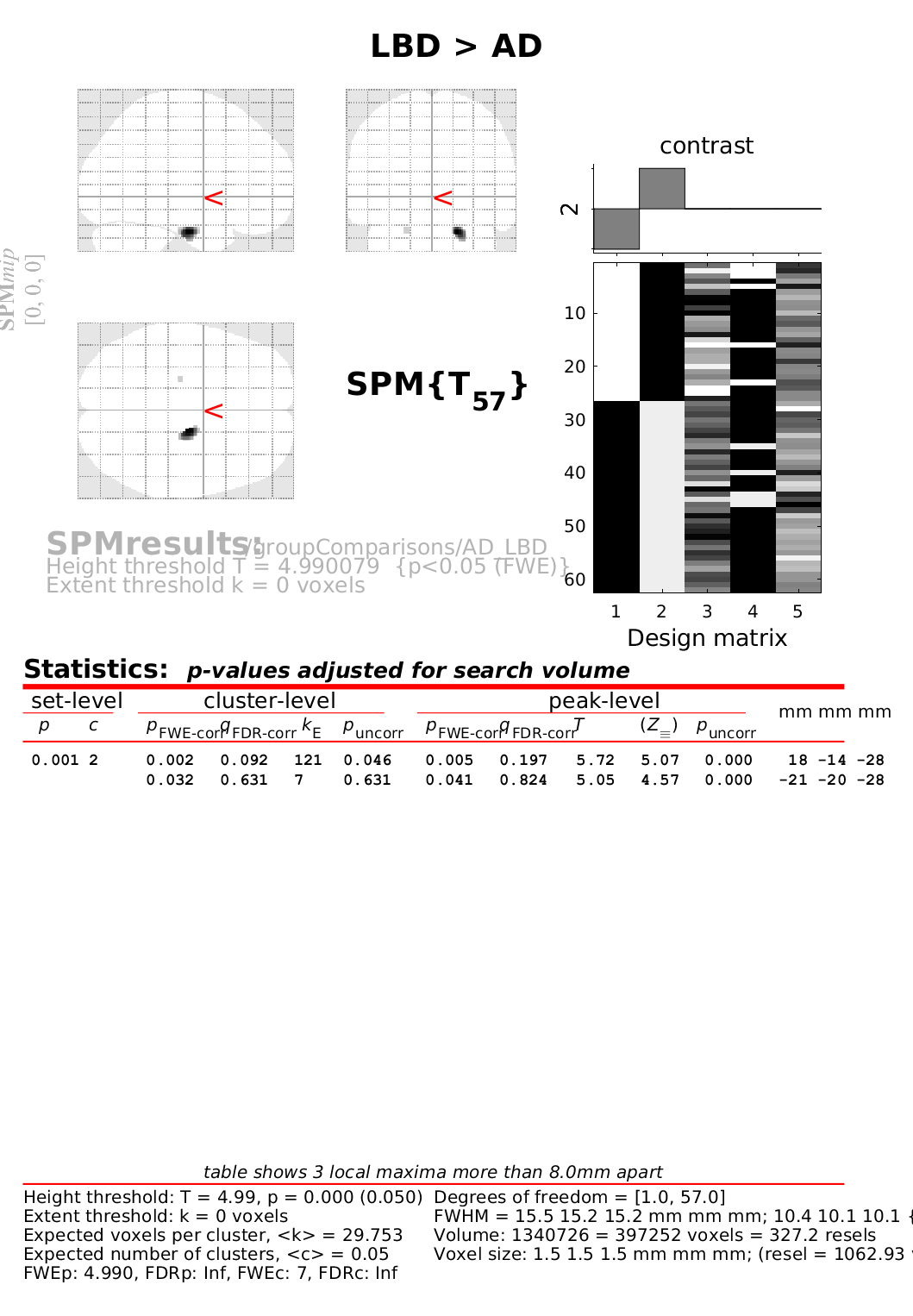


Figure S4: Decrease in GM volume in AD compared to LBD, significant clusters are shown for p<0.05 (FWE corrected).
